# Neuroevolution gives rise to more focused information transfer compared to backpropagation in recurrent neural networks

**DOI:** 10.1007/s00521-022-08125-0

**Published:** 2022-12-17

**Authors:** Arend Hintze, Christoph Adami

**Affiliations:** 1 Department for MicroData Analytics, Dalarna University, Falun, Sweden; 2 Department of Microbiology and Molecular Genetics, Michigan State University, East Lansing, USA

**Keywords:** Recurrent neural network, Transfer entropy, Computation, Memory

## Abstract

Artificial neural networks (ANNs) are one of the most promising tools in the quest to develop general artificial intelligence. Their design was inspired by how neurons in natural brains connect and process, the only other substrate to harbor intelligence. Compared to biological brains that are sparsely connected and that form sparsely distributed representations, ANNs instead process information by connecting all nodes of one layer to all nodes of the next. In addition, modern ANNs are trained with backpropagation, while their natural counterparts have been optimized by natural evolution over eons. We study whether the training method influences how information propagates through the brain by measuring the transfer entropy, that is, the information that is transferred from one group of neurons to another. We find that while the distribution of connection weights in optimized networks is largely unaffected by the training method, neuroevolution leads to networks in which information transfer is significantly more focused on small groups of neurons (compared to those trained by backpropagation) while also being more robust to perturbations of the weights. We conclude that the specific attributes of a training method (local vs. global) can significantly affect how information is processed and relayed through the brain, even when the overall performance is similar.

## Introduction

Natural brains consist of neurons that relay information from the sensors, perform computations on those states, and ultimately activate motor neurons that control muscles. This powerful paradigm has been used as a template to design Artificial Neural Networks (ANNs) [1] since 1943 [2]. However, many aspects of the most powerful ANNs today (for example, Deep Convolutional Neural Networks [3]) differ from the architecture of biological brains in important ways, which has led some to question their relevance in the search for Artificial Intelligence [4, 5].

Among the features that distinguish biological brains from ANNs (besides the sheer number of neurons) are network structure, information encoding, and training method. For example, while biological neural networks are sparsely connected, ANNs typically connect all neurons in one layer to the neurons in an adjacent layer. While convolutional ANNs are almost exclusively feedforward, biological networks have significant connections across layers (forwards and backwards). Even though in the original architecture of ANNs designed by McCulloch and Pitts the neurons were digital (Boolean) and connected to other neurons via Boolean logic implemented using an adaptive threshold, ANNs today usually use continuous variables that are connected in a weighted manner to other such neurons via an activation function (for example, a sigmoid).

In order to reach high-performance levels, the set of weights is optimized using a backpropagation algorithm using gradient descent (see, e.g., [3]). Few studies today have attempted to characterize the impact of these choices on the performance of the network [6, 7], much less on the manner in which information propagates through it. Because a large fraction of the work carried out in Machine Learning is classification, most emphasis has been placed on optimizing the representation of information in networks rather than information flow. In biological brains that control behaving animals, information flow from the sensors through the modules of the brain toward the neurons that actuate muscles is crucial, however [8]. In particular, it is important that relevant signals do not interfere with irrelevant ones and can be processed and integrated effectively “downstream”. Ultimately, such signals must eventually be accessible to only a small group of neurons to drive reliable responses in a postsynaptic network. Which design decisions of ANNs facilitate information processing and flow is currently not known. Here, we study the impact of a single network attribute on information processing (the method of optimization) on a single type of network: a recurrent neural network (RNN). In particular, we study whether and how information flow is affected by either using backpropagation or neuroevolution [9] to train such networks on a simple behavioral task involving the classification of a visual signal. Focusing on information flow in the network shifts our attention away from performance as the only figure of merit of ANNs, toward an understanding of the algorithmic properties of the solution.

Backpropagation is the most common method to train ANNs and owes its fame in part because its discovery [10, 11] led to a resurgence in ANN research as it enabled learning the exclusive OR function. While there is no evidence whatsoever that such a training method is used in biological brains, an alternative method that optimizes weights using a Genetic Algorithm (GA) via *neuroevolution* has a biological equivalent. These two approaches to optimization are fundamentally different, as we now briefly discuss.

**Fig. 1:**
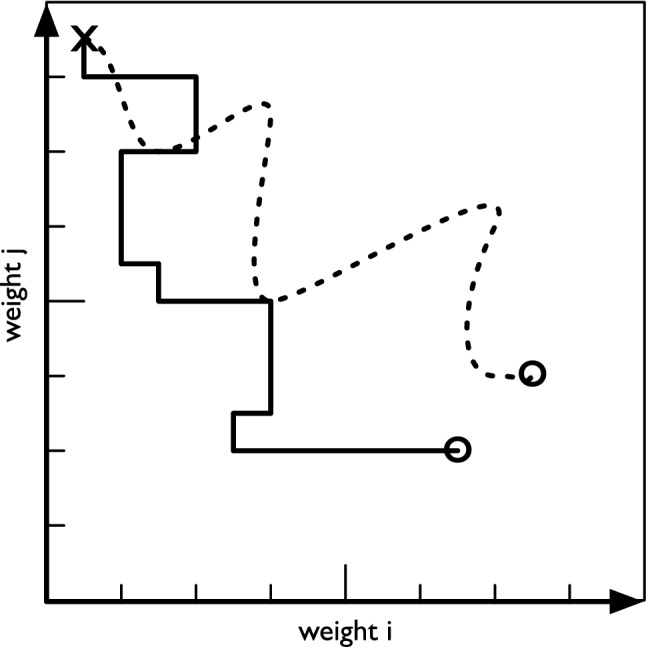
Illustration of how different optimization processes move the candidate solution through solution space. For ease of visualization, imagine a very small neural network with only two weights *i* and *j*, which creates a solution space defined by the two axes *i* and *j* (obviously this could be generalized to a higher-dimensional space). From a starting location X, due to the discrete nature of the mutations, a GA moves the best solution in a population along the axes in a step-wise manner (solid line). In contrast, backpropagation changes all weights simultaneously, resulting in a different, smoother trajectory (dashed line). If the energy landscape of the solution space is rugged, the different processes could give rise to different endpoints (O)

**Fig. 2:**
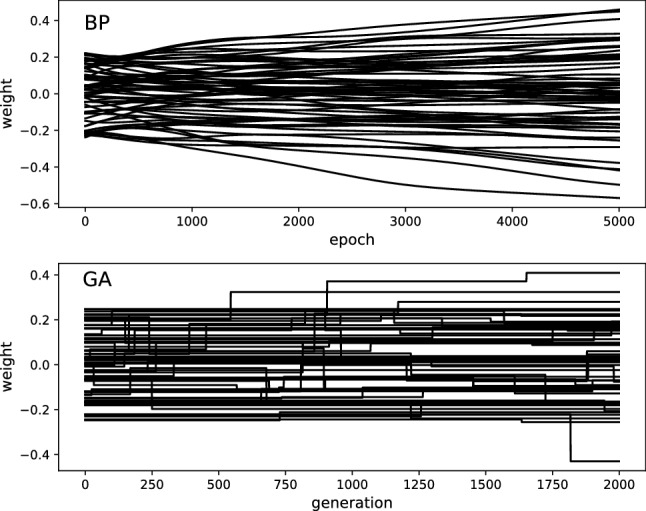
How different optimization methods change the weights of the recurrent layer of an RNN during training. The top panel (BP) shows the weights changed by backpropagation over 5000 epochs. The bottom panel (GA) shows how weights are changed by a mutational operator during optimization using a genetic algorithm. In both cases, a single example of an RNN which attained perfect performance is shown. Weights were seeded using the default Xavier weight initialization [19]

For backpropagation, the difference between expected outputs and actual outputs is computed and then consecutively applied from the last to the first layer to compute a gradient that determines the “direction” (in a high-dimensional space) in which each weight should change. As a consequence, every time the weights of the neural network are changed, they all move toward some optimum in concert. In contrast, a GA will test different candidate solutions in a population for performance and allows better-performing solutions to propagate into the population of the next generation. At that point, mutations are applied to only a few of the weights in a candidate solution, and the process is repeated. Those mutations that convey the largest performance increase have the highest chance of finding themselves in the next generation. For a more intuitive analogy, let us imagine that the weights of the neural network form a multidimensional vector space with one dimension per weight. Backpropagation moves the solution through this space along vectors determined by the gradient of the error function, while the mutations applied in a GA move the solution along only one or a few dimensions at a time (see Fig. 1). While each mutation itself is undirected, the aggregate of selected mutations moves the population of candidates toward higher fitness (for an illustration of the outcome, see Fig. 2).

In neuroevolution, each change to a weight that conveys a benefit is selected with a likelihood proportional to that benefit. While weights still act together to create a fit network, one might expect much less interdependence between weights. We know that in biological brains, neurons are sparsely connected, leading to the assumption that the majority of existing connections are important for information processing. Because a GA directly selects for functionality, it is possible that neuroevolution will tend to produce more sparsely connected networks also, leading to a few (sparse) weights that contribute strongly to functionality, with many others that play no role in function whatsoever.

Since only recurrent neural networks store information in recurrent states, we focus on this type of network here. We previously investigated how representations–internal models of the environment–are stored in evolved computational systems [12, 13]. In humans [14–16] as well as in computational models such as Hierarchical Temporal Memory [17] and Markov Brains [12], these memories are sparsely distributed, meaning that small groups of neurons carry the information associated with complex concepts [18]. Preliminary evidence suggests that memories in RNNs appear to be much more distributed over nearly *all* the nodes of the network [13]. The weight matrix connecting all nodes in a recurrent layer controls how information is stored. If sparsely filled with few weights, the connection matrix might result in more sparsely distributed representations. Conversely, if the resulting weight matrix is fully connected, information might be much more distributed over all nodes. Thus, how information is stored among nodes is determined in part by how information flows through the network.

We will assume by default that the computations carried out by ANNs optimized by different methods should, in principle, be similar if the attained level of performance is similar. It is less clear whether we also should expect that information is stored, processed, and transferred between the neurons in the same way. We can test whether this is so by describing information transfer in the cognitive process mathematically [20–22], using information theory [23, 24]. One method to quantify how information is processed and relayed through nodes is *transfer entropy* (TE) [25, 26].

Transfer entropy is the information-theoretic version of “Granger causality” [27], and reduces to it for auto-regressive processes. Unlike Granger causality, transfer entropy can accurately quantify causality when signals are nonlinear. A related measure is the concept of “directed information,” but since transfer entropy is an upper bound to directed information [28], it is sufficient to study TE. While TE does not always correctly quantify information flow (as we will discuss in more detail below), more complex measures such as “partial information decomposition” [29] are far more costly to evaluate and are unlikely to reveal fundamental differences not already revealed by TE.

The transfer entropy concept has previously been used to study the structure of representations (memory) [30–32]. Simply put, TE measures how much the future state of one neuron or node of a cognitive network is predicted by the state of another node, as well as the node’s own temporal history. However, as previously observed [33], transfer entropy is not a perfect measure by any means. For example, when considering an XOR (exclusive OR) gate, computing the information transferred from one of the inputs of the XOR gate to the output, we find TE to vanish (even though the information is transferred) as the output of this gate is fully determined only if the other input is known as well. This is due to the *polyadic* relationship engendered by the XOR operation, where more than two variables are necessary to determine an outcome. Shannon mutual entropy, however, can only be measured properly for *dyadic* interactions [34], and the TE is, at its heart, composed of Shannon mutual (and conditional) entropies.

While there are a number of other caveats that need to be taken into account when computing TE from finite and noisy data sets (see, e.g., [35]), when applied properly, TE allows us, in principle, to characterize the flow of information in computational cognitive system. For example, Tehrani and Adami [36] studied a type of recurrent neural network known as Markov Brains [37] that were optimized to perform two different cognitive tasks using a Genetic Algorithm. Much like the networks of McCulloch and Pitts [2], the binary logic gates of these artificial brains connect input nodes of the network to hidden nodes, which in turn connect to each other and output nodes. The connections, as well as types of logic gates are specified by a genome that experiences mutations during the evolutionary process. The connections between nodes define a connectivity matrix *C*. When using transfer entropy between each pair of nodes in the Markov Brain to determine the information flow matrix *T*, they find these matrices to be similar (see Figure 6 of Ref. [36]), leading them to conclude that connectivity alone significantly (but not perfectly) predicts information flow in these recurrent neural networks.

In earlier work, we found instead that the transfer entropy between pairs of nodes in recurrent networks does *not* correlate with the importance of the weight connecting them and that the type of optimization algorithm (be it backpropagation or neuroevolution) has no bearing on the relationship between information flow and edge strength [38]. While the Markov Brains investigated in [36] and the traditional RNNs studied in [38] are sufficiently different to explain this observed difference, it is still odd that information flow through a pair of neurons is unaffected by the weight between them. Here we study whether this conclusion rests on restricting the analysis to only pairs of neurons by studying the transfer entropy between *sets* of neurons instead. Such an analysis is bound to be more accurate if information is conveyed predominantly by such sets of neurons (as we might expect in recurrent networks), in particular when the TE through pairs of neurons vanishes due to logic gates that encrypt information.

## Material and methods

### Categorical perception task

The overlap between cognitive science and artificial intelligence research seems to shrink further, as the cognitive tasks and research foci in both fields seem to drift apart [39]. This makes it harder to find tasks that are interesting to both domains. In addition, the task must allow us to test the correlation between TE and connectivity in RNNs trained in two different ways–backpropagation or neuroevolution. Thus, the task needs to be simple enough so that either method can find an optimal solution. At the same time, the task needs to be difficult enough that information must be processed in a complex fashion.

**Fig. 3:**
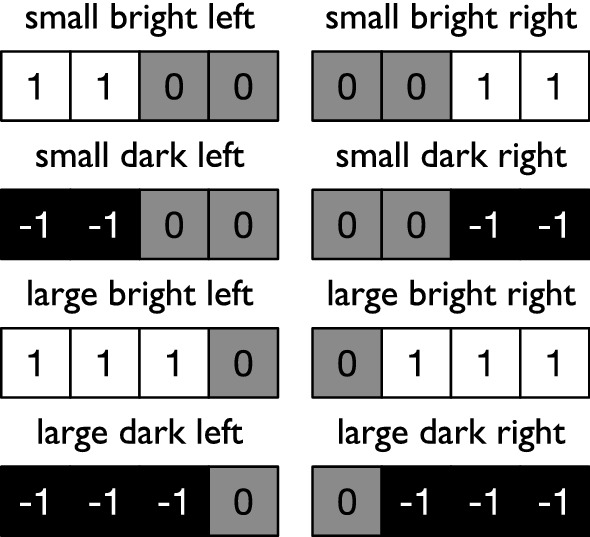
illustration of the eight possible block configurations. Blocks can have three properties: small or large, bright or dark, left or right. A large block occupies three squares while small blocks occupy two. The brightness is indicated by 1 (bright) or $-1$ (dark). A brightness value 0 indicates an empty slot

Consequently, as in [38], the task we use here is similar to active categorical perception (ACP) [40, 41]. This task is similar to a robotics control task where objects need to be recognized properly for the robot to function. Here the identity of an object has to be ascertained visually, collecting data over multiple time points, and the decision is rendered behaviorally (as in a monkey pulling a particular lever). In this abstracted version of ACP, successfully completing the task requires both retention and integration of information. Here, two different blocks are presented to the RNN. The blocks can be large or small and either to the left or to the right of the vision field. They are further either colored bright or dark, as inputs 1.0 or $-1.0$ to the input (retina) layer of the RNN (see Fig. 3 showing all eight possible input patterns).

**Fig. 4:**
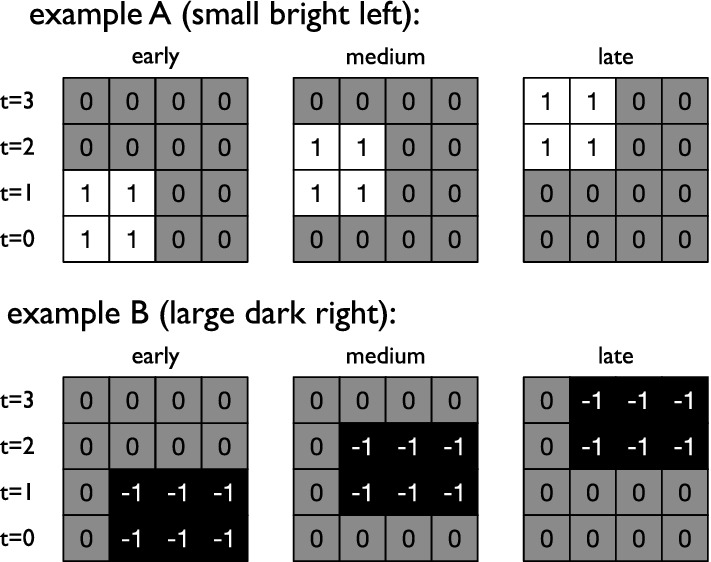
Two examples of possible patterns presented to the RNN. Each possible pattern is fed into the RNN sensory layer one line at a time, over four time points. In example A (top), a small bright block on the left of the viewing field is shown. In example B (bottom) a large dark block on the right side of the viewing field is presented

The blocks are presented twice to the RNN on succeeding time steps over the course of four total time steps. This implies that blocks can be shown early, at an intermediate time, or late to ensure that the RNN cannot simply rhythmically synchronize to the signal and has to store information about the block at least until the fourth time step. In the early case, the blocks are shown a time point $t=0$ and $t=1$, while at the other two time points an empty line (all values 0) is shown. When the block arrives late, two empty lines are shown during time point $t=0$ and $t=1$, followed by the block at time points $t=2$ and $t=3$. For the intermediate condition, the block is shown at time points $t=1$ and $t=2$, while an empty line is shown at the remaining time points (Fig. 4 shows a typical example).

### Training of recurrent neural network

**Fig. 5:**
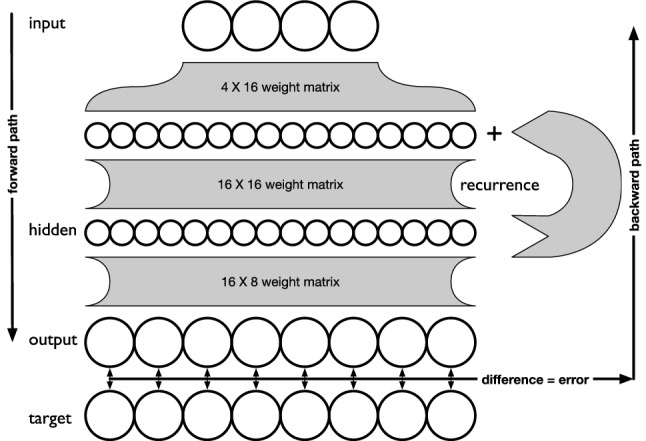
Schematic illustration of the RNN architecture. The RNN receives four inputs that run through the first layer to become amplified to 16 nodes, which is the size of the recurrent layer. The recurrent layer states are added and processed through a $16\times 16$ weight matrix to form the recurrent layer states. Those states are then ‘recurred’ to the next time step while at the same time propagated forward to form the eight outputs. Each node has a summation aggregation function and a hyperbolic tangent threshold function. Each node also has a bias, not shown in the figure. In the case of backpropagation, inputs enter from the top and follow the path illustrated. The computed output is then compared with the expected target, the difference is computed and defines the error. The error propagated backwards through the network following the backwards path to compute gradient and to change the weights of the network accordingly

The recurrent neural network has four input (sensory) nodes, followed by a recurrent layer of size 16 using an Elman architecture [42], feeding into an output layer of 8 nodes, one for each of the eight possible categories (see Fig. 5). Note that this choice of output implies that we have (for the sake of simplicity) substituted the behavior of catching or not-catching a particular block by its categorization instead.

Every node in the RNN is also characterized by a *bias*, which can be thought of as an additional weight, as it is affected by either optimization method exactly like a weight. At inception, networks are populated with random weights using the Kaiming initialization method [43]. We use the summation of inputs to all activation functions, which here is the hyperbolic tangent function. When using backpropagation for training, we compute the loss as the mean squared error and facilitate backpropagation by the stochastic gradient descent (SGD) optimizer from the *pytorch* library [44] and computed the mean squared error as the loss function. When we use a GA for optimization, we create a population of 100 random RNNs and select to propagate a mutated copy of each genome encoding weights and biases into the next generation with a likelihood proportional to the performance (see Eq. 3) of the RNN. Each weight in the genome experiences a chance of 0.01 to be modified by a random number drawn from a normal distribution with a vanishing mean and a variance of 0.1.

As in [38], to assess the performance of each RNN in order to calculate a fitness function for the GA, we calculate the difference $D=O-A$ between the outputs *O* and all expected (correct) answers *A*, so as to compute the squared error

1 $$\begin{aligned} E=\sum \limits _{i=0}^{n}\sum \limits _{j=0}^{m}D_{i,j}^{2}. \end{aligned}$$

This error is then normalized and transformed into a fitness

2 $$\begin{aligned} w=1.0-\frac{E}{nm} . \end{aligned}$$

Finally, this fitness is multiplied by the total number of accurate classifications *a* and raised to an exponent to yield the function

3 $$\begin{aligned} W=1.5^{wa} . \end{aligned}$$

Note that the dimensions of the output or expectation matrix are defined by the number of inputs to classify ($n=24$) and the number of possible classes ($m=8$). The value of the base used in the transformation to an exponential function determines the strength of selection and is the result of trial and error. The chosen value depends on the particular task being optimized and should result in a fitness function that finds optimal solutions sufficiently fast while preserving variation. We found that over 2,000 generations of evolution were necessary to evolve 50 independent populations of 100 RNNs to all have perfect performance at the end. For the backpropagation runs, 5000 epochs were necessary.

### Transfer entropy

The transfer entropy $\textrm{TE}_{i\rightarrow j}$ from node *i* to node *j* can be written in terms of a shared conditional entropy using the state variables $X_t^{(i)}$ and $X_t^{(j)}$ as

4 $$\begin{aligned} \textrm{TE}_{i\rightarrow j}= &\,H(X^{(j)}_{t+1}:X^{(i)}_{t}|X_t^{(j)})\nonumber \\= & \sum _{x_{t+1}^{(j)},x_{t}^{(j)},x_t^{(i)}}\quad p(x_{t+1}^{(j)},x_{t}^{(j)},x_t^{(i)})\log _2\frac{p(x_{t+1}^{(j)}|x_t^{(i)},x_t^{(j)})}{p(x_{t+1}^{(j)}|x_t^{(j)})}. \ \ \ \end{aligned}$$

In Eq. (4), $x_t^{(i)}$ refers to the state of variable $X^{(i)}$ at time *t*, while $p(x_t^{(i)})$ is the probability to find variable $X^{(i)}$ at time point *t* in that state. Further, standard notations for conditional probabilities apply. While in general, the variables at time *t* may include the history of that variable up to time *t*, here we only consider the most recent state (Markovian dynamics). Furthermore, to obtain the joint probability $p(x_{t+1}^{(j)},x_t^{(i)},x_t^{(j)})$, the variables are sampled over the entire time series for all possible 8 environments, under all three temporal conditions presented to the RNN.

For both training conditions (backprop and neuroevolution) we record the state-to-state transitions for each of the 50 RNNs with perfect performance, to obtain $p(x_{t+1}^{(j)},x_t^{(i)},x_t^{(j)})$ for the recurrent nodes, which is sufficient to compute Eq. (4). As the states $x_t^{(i)}$ are continuous at the outset, we discretize them using the median value as a threshold. Values above the median are set to 1; those below the median are set to 0. This discretization ensures that the entropy $H(X_t^{(i)})$ for each node is maximal [13]. After the discretization, the transfer entropy between all node pairs is computed using the Python library *Smite* [45] for each RNN performing perfectly after optimization.

The measure of transfer entropy (4) can easily be extended to not only consider single nodes *i* and *j*, but instead, between sets of source nodes ${\mathbb {S}}$ and target nodes ${\mathbb {T}}$. However, those sets cannot share a node (they must be disjoint), and neither set can be empty. When computing the transfer entropy between sets $\mathrm TE_{{\mathbb {S}}\rightarrow {\mathbb {T}}}$ the joint random variables from all nodes in ${\mathbb {S}}$ and ${\mathbb {T}}$ have to be computed. $\mathrm TE$ was calculated based on that data using our own analysis software.

### Node-weight perturbations

As in [38], we determine the importance of a connection between two neurons by perturbing the optimized weight between them. A weight matrix *M* specifies the weights connecting each node to each other node of the recurrent layer. To estimate how important each weight (or a group of weights) is to the performance of the RNN, we perturb node weights by shifting each weight by a random offset *r* at every step of the computation. We choose this perturbation from a uniform distribution with a variable upper bound. Specifically, we perturb the weight connecting nodes *i* and *j* in 21 steps, with an increasing upper bound starting at 0 (no perturbation) and ending at 4.0.

For each of the different noise increments, we record the average relative performance of the network ${\bar{W}}_{i,j}=\langle {\tilde{w}}_{ij}/w^{(0)}_{ij}\rangle$, where ${\tilde{w}}_{ij}$ is the performance of the network under perturbation of the weight between *i* and *j*, and $w^{(0)}_{ij}$ is the “wild-type” (unperturbed) performance (similarly for perturbation of groups of neurons). If a weight is not involved in the function of the network, we expect the performance of the network as a whole to be unaffected. On the contrary, if a weight’s value is crucial for the network’s function, performance will drop. We can thus define the *importance* *I* of the edge as:

5 $$\begin{aligned} I_{ij}=1.0-{\bar{W}}_{i,j}\; . \end{aligned}$$

**Fig. 6:**
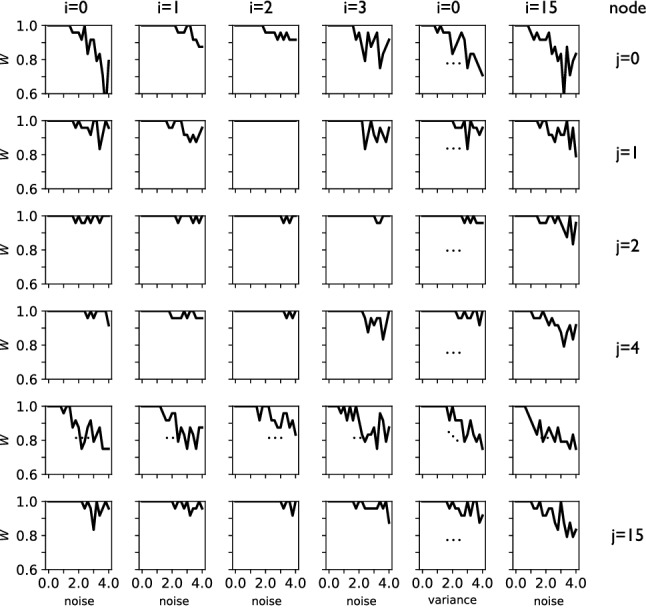
Average performance loss ${\bar{W}}_{i,j}$ (performance under noise divided by unperturbed performance) for each weight connecting node *i* and *j*, for increasing levels of noise. At each computational update, the weight is modulated by adding a random number drawn from a uniform distribution $U\in [0,r]$. For example, the weight connecting node $i=15$ with node $j=0$ (top right corner) has a rapid drop in performance when the noise increases, indicating that this weight is important. The weight connecting node $i=2$ to node $j=1$, on the other hand, is not important since no level of noise is changing the performance of the network. In this particular illustration the network is optimized using neuroevolution, but similar patterns occur quite generally

Figure 6 shows an example of this calculation, which depicts the average performance loss ${\bar{W}}_{i,j}$ for different combinations of *i* and *j* (rows and columns). In each quadrant, we can see how performance deteriorates as the noise is increased for some edges while staying constant for others.

When testing for the importance of connections between *sets* of nodes (specifically those measured by $\mathrm TE_{{\mathbb {S}} \rightarrow {\mathbb {T}}}$) multiple weights have to be perturbed at the same time. In that case, all weights connecting the nodes of ${\mathbb {S}}$ to ${\mathbb {T}}$ experience the same noise.

## Results

As in [38], we trained RNNs using a GA or using backpropagation in 50 independent replicates until they all performed optimally. Then, for the recurrent layer, we calculate the transfer entropy between sets of notes ${\mathbb {S}}$ and a single target neuron *j*, along with the average performance loss (perturbed performance relative to unperturbed) when the weight connecting the nodes in set ${\mathbb {S}}$ and *j* is subjected to weight perturbation (to calculate the importance of the weight). The results in [38] are included here as the case where the set ${\mathbb {S}}$ contains only a single neuron. As a reminder, if TE is measured between pairs of nodes only, we did not find a correlation between transfer entropy and the importance of nodes. We also did not find a difference between the optimization methods.

### Beyond one-to-one connections

We now test whether the expected correlation is absent because the information in RNNs is carried by groups of neurons, so perturbing a single neuron may affect performance but without affecting the flow through a single neuron. As observed, for example, in groups of neurons encoding decisions in macaque monkeys [46], larger groups of neurons can be found that together carry the information processed by the network, and that information decreases as neurons are removed from such a group. Indeed, it is known that when logic is implemented using multi-variate logic (multiple-in–multiple-out-gates), the information between any *two* of those variables can appear to be low, or even absent [36].

To test for this eventuality, rather than computing $\mathrm TE_{i\rightarrow j}$, where *i* and *j* are single nodes, we should compute the transfer entropy from a set of source nodes ${\mathbb {S}}$ to a set of target nodes ${\mathbb {T}}$ ($\textrm{TE}_{{\mathbb {S}} \rightarrow {\mathbb {T}}}$), while ensuring that no element of ${\mathbb {T}}$ is an element of ${\mathbb {S}}$ and neither set is empty. Unfortunately, the number of possible sets one can test grows exponentially with the number of nodes in the set, precluding an exhaustive analysis for any but the smallest systems. However, we can test if increasingly larger sets convey more information while also becoming more important to function by sub-sampling from the space of possible sets, and limiting the target set ${\mathbb {T}}$ to only one node.

**Fig. 7:**
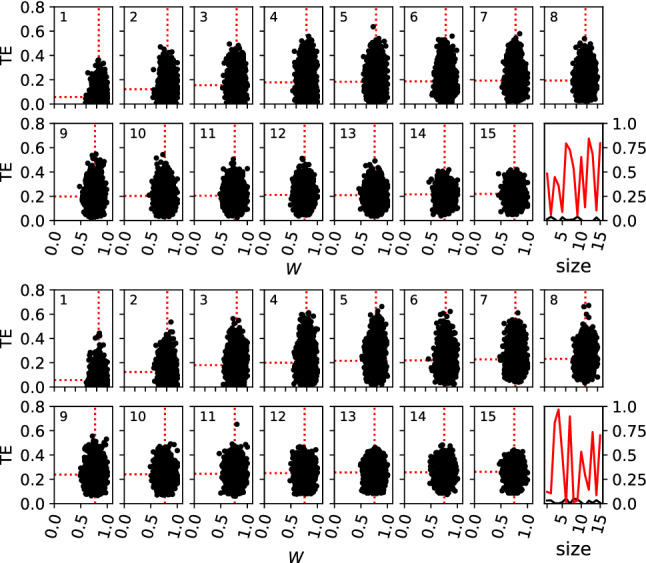
Correlation between transferred information TE (in bits) and importance *I* as measured by Eq. (5). The top panel shows the results from RNNs trained by BP, and the lower panel is the same for RNNs trained by a GA. The number on each sub-panel 1–15 refers to the size of the source set ${\mathbb {S}}$ used. Each sub-panel 1–15 shows the $\textrm{TE}_{{\mathbb {S}} \rightarrow {\mathbb {T}}}$ on the *y* axis, and the average fitness loss ${\bar{W}}$ for each measured subset as the *x*-axis. All 50 perfect performers were sub-sampled 100 times for each size of ${\mathbb {S}}$. Average performance loss and average $\mathrm TE$ are shown as dotted red lines. The last sub-panel (16) shows the p-value of Spearman’s rank correlation coefficient (as we cannot assume a normal distribution for either axis) in red, and the respective correlation coefficient in black for each possible size of the subset

With an increase in nodes in the source set, it might be possible to better detect a correlation between the importance of weights and the information transferred. An increase in the set size should then result in an increase in the correlation between importance and transfer entropy. However, we do not find stronger correlations to appear: All p-values for Spearman’s rank correlation coefficient between TE and average fitness loss reject the hypothesis that the data is correlated for any of the set sizes studied (see Fig. 7).

When increasing the set size, we expect an increasing loss of performance as an increasing number of weights are perturbed simultaneously. At the same time, we expect an increase in transferred information because larger sets of neurons can carry more entropy. Interestingly, we find that with increasing set sizes, RNNs optimized by a GA experience less functional loss due to perturbation than their BP-optimized counterparts, meaning they are more robust to perturbation. At the same time, we observe a greater increase in transferred information with set size in RNNs trained by a GA as opposed to those trained by BP.

**Fig. 8:**
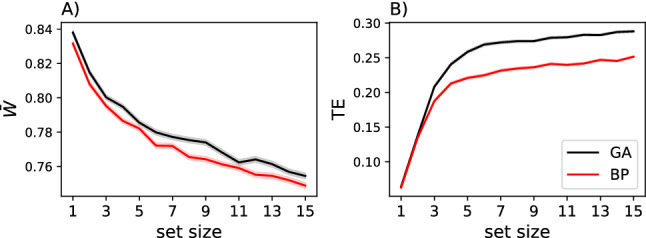
**a** Importance of weights as a function of set size of perturbed weights, for differently trained RNNs (BP in red, GA in black). The averages shown here are taken from the same data presented in Fig. 7. **b** Average $\textrm{TE}_{{\mathbb {S}} \rightarrow {\mathbb {T}}}$ (in bits) for different sizes of ${\mathbb {S}}$ (red are RNNs trained by BP, and black for RNNs trained using a GA). All lines have a red or black shadow underlying indicating the standard error of the respective data point (vanishingly small) (color figure online)

**Fig. 9:**
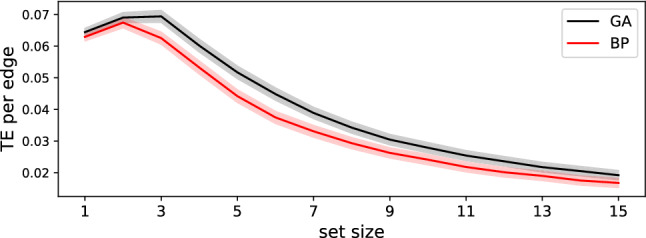
Information transferred per node in sets of nodes, in networks trained using a Genetic Algorithm (GA, black) or backpropagation (BP, red). The shadows behind the solid lines indicate the standard error over the 50 replicate experiments (color figure online)

When normalizing the transferred entropy in a set by the size of the set, we find that in GA-optimized networks sets of size 3 transfer the most information per connection, while in networks optimized with backpropagation, the maximum of information transmission occurs in sets of size 2. This observation might explain the differences in robustness we see in Fig. 8a, as smaller sets of nodes will be more prone to information disruption than larger sets, which can protect information via redundancy. Thus, information transfer in networks optimized by neuroevolution is more focused in the sense that the information density (information transferred per neuron) is higher while at the same time more robust as information is optimally encoded in larger sets, compared to networks optimized by backpropagation (see Fig. 9).

**Fig. 10:**
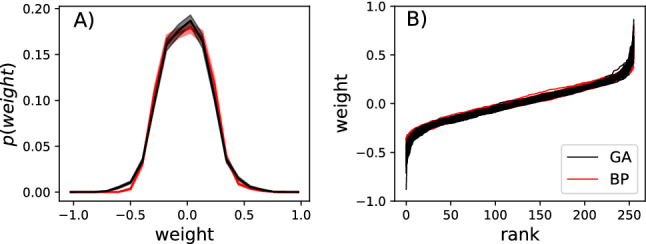
Comparison between weights optimized by a GA (black) and BP (red). Data from all perfectly functional RNNs was aggregated. Panel A) shows the weight distribution for both GA and BP optimized RNNs at the end of optimization. The shadows behind the solid lines indicate the 95% confidence intervals. Panel B) shows the weights for each optimized neural network after they have been sorted from low to high. The rank is plotted on the x-axis, while the weight is shown on the y-axis. Again, GA is in black, BP is in red (color figure online)

We now test whether these observed differences could be due to differences in the mutational operator used during optimization. The GA randomly changes weights one by one (and probabilistically rejects those that do not increase performance), while the gradient descent optimizer smoothly adjusts all weights simultaneously according to a calculated gradient. This algorithmic difference could lead to vastly different weight distributions, which in turn could cause the observed differences in noise robustness and information transfer. However, both distributions, even though they are significantly different (Kolmogorov-Smirnov test difference 0.0189 with a $P=0.0198$) have a similar shape and range (see Fig. 10a), averaged over all replicates). We find that the weight distribution obtained from optimization via a Genetic Algorithm shows an excess of zero weights compared to the distribution obtained via backpropagation, suggesting that GAs are more efficient in creating sparser networks by turning off connections altogether.

Because aggregated probability distributions tend toward universality due to the central limit theorem, we further tested the weight distribution sorted by rank and then displayed them individually for each optimization attempt. We find almost no differences in that kind of visualization (see Fig. 10b).

## Discussion

The difference between backpropagation and optimization using a GA with respect to performance, ease of use, and efficiency has been tested extensively before [47–49]. Recently, the existence of modern libraries supporting both methods has made it significantly easier to compare those methods directly.

To the best of our knowledge, this is the first study of potential differences in structure and/or function of the resulting networks due to differences in the optimization algorithm. Interestingly, while both methods produce highly functional systems, we previously found no differences in how information is processed in these networks, judging by the correlation between information flow and weight importance for pairs of nodes [38]. Here we went beyond the pairwise constraint and tested how sets of nodes transfer information. Even though we might have expected that larger groups of neurons that carry more information would also be more prone to information disruption from weight perturbation, we found this not to be the case. Instead, we found that encoding information into larger groups of neurons not only increases the information density but also provides robustness to noise via redundancy, with networks trained via a Genetic Algorithm doing this more effectively.

We used standard backpropagation and GA for optimization. For backpropagation, stochastic gradient descent using a mean squared error loss function applied to a simple RNN using Elman architecture and hyperbolic threshold functions was used. Similarly, the GA used a small population and applied simple point mutations to the weights without recombination. Selection was made using roulette wheel selection without further elitism or other more advanced methods. Of course, different optimizers, loss functions, selection methods, or network architectures might affect the outcome. For example, dropout [50], since it influences which weights are used or not used during training, might influence the results. However, for a proper comparison between backpropagation and a GA, dropout should then also happen during the GA’s optimization process. We would still expect to observe a similar difference between both optimization processes, regardless of hyper-parameter selection or architecture, even though a more thorough investigation of hyper-parameter selection and their effects on information propagation might be revealing.

Previous studies of information coding in the brain of macaque monkeys have shown that information is indeed carried in groups of neurons in a redundant manner so that the removal of neurons from the group reduces the information carried by the remaining group only gradually [46]. This is in contrast to the possibility of cryptic encoding of information into groups, where information can abruptly disappear once a set of key neurons has been removed from the group. This finding suggests that cryptographic encoding plays no role in information encoding in RNNs, presumably because the cryptographic functions (XOR and XNOR) are difficult to achieve using this network architecture, while cryptographic encoding is common in different architectures [36].

Of course, the active categorical perception task we studied here is only one of many possible tasks that biological and computational neural networks are applied to. It is certainly possible that other tasks might highlight different computational differences between network training methods. The important observation here is that the methodology we presented is capable of detecting such differences. Also, topological differences in recurrence, such as those found in LSTM (Long short-term memory) or GRU networks [51, 52] could, in principle, affect how information flows in neural networks, which is another interesting direction to pursue.

When comparing network training methods, we only compared a single backpropagation optimizer (ADAM [53]) to only one way of implementing neuroevolution (an asexual Genetic Algorithm with fitness-proportional selection). Details of the optimization method could affect our conclusions. For example, using genome recombination [54] in addition to point mutations could affect how information is encoded, as could the use of indirect encoding methods [55].

We did not test here whether the observed differences in information density and optimal size of information-carrying sets have consequences for the evolvability of these networks. It is conceivable that changing environments or circumstances require a quick re-direction of information flow, which requires the information to be both accessible and encoded into smaller sets. We also did not test how the variables we measured (information flow and robustness) change when the target set size is varied as well. We plan to address this in future work.

We conclude that backpropagation pursues functional optimization without regard to a network’s structure, while optimization via neuroevolution attempts both: an optimization of function, as well as an optimization of the network’s structure in support of that function. The work also suggests that fully-connected recurrent neural networks are likely an inferior starting point for optimization because much of the optimization is concerned with reducing the influence of unwanted connections, suggesting that sparse networks trained via neuroevolution might hold an advantage over a large class of ANN architectures used in the literature.

## Data Availability

The datasets generated during and/or analyzed during the current study are available in the github repository including the scripts used for analysis, https://github.com/Hintzelab/NCAA-D-21-01407R2.
